# A panel of janus kinase inhibitors identified with anti-inflammatory effects protect mice from lethal influenza virus infection

**DOI:** 10.1128/aac.01350-23

**Published:** 2024-03-12

**Authors:** Yang Yu, Si Chen, Haonan Zhang, Yuanyuan Duan, Zhuogang Li, Lefang Jiang, Weihua Cao, Qun Peng, Xulin Chen

**Affiliations:** 1 Institute of Medical Microbiology, College of Life Science and Technology, Jinan University, Guangzhou, China; 2 State Key Laboratory of Virology, Wuhan Institute of Virology, Chinese Academy of Sciences, Wuhan, China; IrsiCaixa Institut de Recerca de la Sida, Badalona, Barcelona, Spain

**Keywords:** influenza A virus, janus-kinase (JAK) inhibitor, anti-inflammatory, oclacitinib maleate, mouse model

## Abstract

Influenza remains a significant threat to public health. In severe cases, excessive inflammation can lead to severe pneumonia or acute respiratory distress syndrome, contributing to patient morbidity and mortality. While antivirals can be effective if administered early, current anti-inflammatory drugs have limited success in treating severe cases. Therefore, discovering new anti-inflammatory agents to inhibit influenza-related inflammatory diseases is crucial. Herein, we screened a drug library with known targets using a human monocyte U937 infected with the influenza virus to identify novel anti-inflammatory agents. We also evaluated the anti-inflammatory effects of the hit compounds in an influenza mouse model. Our research revealed that JAK inhibitors exhibited a higher hit rate and more potent inhibition effect than inhibitors targeting other drug targets *in vitro*. Of the 22 JAK inhibitors tested, 15 exhibited robust anti-inflammatory activity against influenza virus infection *in vitro*. Subsequently, we evaluated the efficacy of 10 JAK inhibitors using an influenza mouse model and observed that seven provided protection ranging from 40% to 70% against lethal influenza virus infection. We selected oclacitinib as a representative compound for an extensive study to further investigate the *in vivo* therapeutic potential of JAK inhibitors for severe influenza-associated inflammation. Our results revealed that oclacitinib effectively suppressed neutrophil and macrophage infiltration, reduced pro-inflammatory cytokine production, and ultimately mitigated lung injury in mice infected with lethal influenza virus without impacting viral titer. These findings suggest that JAK inhibitors can modulate immune responses to influenza virus infection and may serve as potential treatments for influenza.

**IMPORTANCE:**

Antivirals exhibit limited efficacy in treating severe influenza when not administered promptly during the infection. Current steroidal and nonsteroidal anti-inflammatory drugs demonstrate restricted effectiveness against severe influenza or are associated with significant side effects. Therefore, there is an urgent need for novel anti-inflammatory agents that possess high potency and minimal adverse reactions. In this study, 15 JAK inhibitors were identified through a screening process based on their anti-inflammatory activity against influenza virus infection *in vitro*. Remarkably, 7 of the 10 selected inhibitors exhibited protective effects against lethal influenza virus infection in mice, thereby highlighting the potential therapeutic value of JAK inhibitors for treating influenza.

## INTRODUCTION

Influenza virus is one of the most important pathogens in humans, causing considerable morbidity and mortality throughout the year. Despite the widespread use of antivirals in recent years, the mortality caused by influenza has not been significantly reduced (1). The current antiviral drugs for influenza are oseltamivir, zanamivir, peramivir, and baloxavir marboxil. These are all direct-acting antiviral drugs, which are easy to induce drug-resistant variants. In addition, antivirals are only effective during the early stages of infection. The primary cause of patient death is severe pneumonia and acute respiratory distress syndrome (ARDS), caused by the body’s excessive immune response toward the viral infection (2, 3).

While the host’s innate immune system produces interferon and cytokines to fight against the virus, too much cytokine production can result in a harmful “cytokine storm” (4, 5). Deleting or repressing specific cytokines or their receptors can alleviate the illness and reduce mortality (6). For instance, tumor necrosis factor receptor 1 (TNFR1) deficient mice are less susceptible to H5N1 virus infection (6, 7), and treatment with anti-TNF antibodies reduces the severity of illness under influenza or respiratory syncytial virus infection (8). CCR2 antagonist and CCR2 deletion alleviate the pathology and morbidity and protect mice from lethal PR8 infection, though the virus titer was even higher in the treated mice (9, 10). Details about the biological functions of most cytokines are still needed (11).

During influenza virus infection, several types of cells, such as alveolar epithelial cells, endothelial cells, and macrophages, produce pro-inflammatory cytokines/chemokines. The Janus kinase-signal transduction and activator of transcription (JAK-STAT) signaling pathway regulates their expression and is implicated in the pathogenesis of inflammatory and autoimmune diseases (12–14). The pathways include four JAKs and seven STATs in mammals, which regulate the transcription of target genes (12, 15, 16). Multiple interferons and cytokines signal through the JAK-STAT pathway, thus making the latter a promising target for clinical interventions.

Currently, 25 JAK and 3 STAT inhibitors are being tested in various clinical trials, from rheumatoid arthritis (RA), psoriasis, and many other inflammatory diseases to multiple cancers. Although oclacitinib is only approved for the treatment of allergic dermatitis in canines, three JAK inhibitors, including ruxolitinib, tofacitinib, and oclacitinib, have been approved by the US Food and Drug Administration (FDA) for clinical use (17). However, no specific immunomodulatory agents are available for treating severe influenza virus infection. Classical anti-inflammatory drugs such as corticosteroids and COX-2 inhibitors have been reported to worsen patient outcomes or have no obvious effectivity in the clinic (18–20). Therefore, novel drugs that suppress influenza-induced inflammation are urgently needed.

In this study, we screened a collection of 2,137 drugs using a monocytic U937 cell assay system (21). A panel of JAK inhibitors have been identified to suppress the production of three pro-inflammatory cytokines that are upregulated in cases of severe influenza. Most JAK inhibitors tested in an influenza mouse model showed potent therapeutic effects in protecting mice infected with the lethal influenza virus. Based on the *in vitro* and *in vivo* studies, we propose that JAK inhibitors represent a promising class of anti-inflammatory agents for treating influenza virus-induced inflammatory disease.

## RESULTS

### JAK inhibitors were identified as anti-inflammatory agents using a U937 cell model infected with influenza virus

Since animal models are not suitable for high-throughput screening of anti-inflammatory agents, we previously established a U937 cell model to test drugs that reduce proinflammatory cytokines (IP-10, MCP-1, and IL-8) induced by the influenza virus (21). We screened a drug library of 2,137 compounds at 10 µM concentration. Since antiviral agents can lower the production of proinflammatory cytokines by suppressing viral replication associated with cytokine production, compounds with both antiviral and anti-inflammatory activity were excluded from the hits. We found that JAK inhibitors had the highest hit rate (68%) among inhibitors of all categories of drug targets for anti-inflammatory activity. A total of 15 hits among 22 JAK inhibitors were screened out to have anti-inflammatory effects against influenza virus infection.

To further confirm whether the hit JAK inhibitors have anti-inflammatory effects, we tested the concentration-dependent inhibition on the production of each of the three cytokines and determined the concentrations of 50% inhibition and cytotoxicity (IC_50_ and CC_50_, respectively) and the selective index (SI) for each JAK inhibitor. The 15 hit JAK inhibitors were confirmed to inhibit the production of IP-10, IL-8, and MCP-1, with a selective index of more than 10 in the U937 cell-based model (Table 1). We tested 10 of the hit JAK inhibitors (highlighted in bold chosen for the *in vivo* study) for possible antiviral activity at serially diluted concentrations up to 200 µM. The results showed that they all exhibit no antiviral activity against the influenza virus (data not shown), suggesting that their anti-inflammatory activity is not a result of inhibition of virus replication. Of note, nearly all of the identified JAK inhibitors have an IC_50_ at the nanomolar range, and most of them have a (SI) of more than 1,000 (Table 1), suggesting that these JAK inhibitors have potent anti-inflammatory activities in reducing the production of specific pro-inflammatory cytokine/chemokine induced by influenza virus infections.

**TABLE 1:** Identification of JAK inhibitors as anti-inflammatory agents using U937 cells infected with influenza virus^*a*^

Inhibitors	Targets	CC_50_ (µM)^*b*^	IC_50_ (µM)^*c*^			SI^*d*^		
			IL-8	IP-10	MCP1	IL-8	IP-10	MCP1
**AZ 960**	JAK2	6.0	<0.005	0.01	<0.005	>1,200	600	>1,200
**AZD1480**	JAK2	13.5	1.1	1.0	0.3	12	14	45
**Baricitinib**	JAK1/2 and Tyk2	>100	0.07	0.04	0.08	>1,429	>2,500	>1,250
CEP-33779	JAK2	10.5	0.36	0.22	0.24	29	48	44
Cerdulatinib hydrochloride	JAK1/2/3 and Tyk2	3.5	<0.005	0.05	0.02	>700	70	175
Decernotinib	JAK1/2/3 and Tyk2	50	0.02	0.10	0.05	2,500	500	1,000
**Gandotinib**	JAK1/2/3 and Tyk2	26	< 0.005	0.01	<0.005	>5,200	2,600	>5,200
Momelotinib	JAK1/2/3	5.4	0.3	0.48	0.03	18	11	180
**Oclacitinib maleate**	JAK1/2	50	0.05	0.18	0.19	1,000	278	263
**Peficitinib**	JAK2	34.7	0.01	0.02	0.01	3,470	1,735	3,470
**Ruxolitinib**	JAK1/2	100	0.12	0.07	0.03	833	1,429	3,333
**S-Ruxolitinib**	JAK1/2 and Tyk2	100	0.03	0.08	0.06	3,333	1,250	1,667
**Tofacitinib**	JAK1/2/3	>100	0.01	0.02	0.05	10,000	5,000	2,000
**Tofacitinib citrate**	JAK2/3	>100	0.02	0.04	0.04	>5,000	>2,500	>2,500
XL019	JAK1/2/3	26.7	0.51	0.99	0.94	52	27	28

^*a*^ Inhibitors in bold are selected for further studies in animal experiments.

^*b*^ CC50: the compound concentration required to reduce cell viability by 50%, as determined by the CellTiter-Glo Assay (Promega). Data represent the mean of three independent experiments.

^*c*^ IC50: the compound concentration required to inhibit cytokines production by 50%, as determined by Alphalisa assay. Data represent the mean of three independent experiments.

^*d*^ SI, the ratio of CC50/IC50.

### JAK inhibitors protect the mice from lethal influenza virus infection

**Fig 1:**
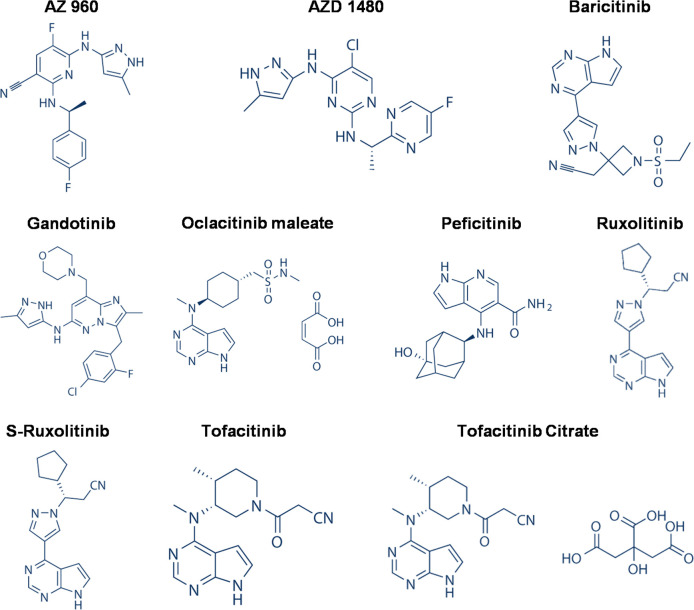
Chemical structures of 10 selected JAK inhibitors that were tested in the *in vivo* experiments.

**Fig 2:**
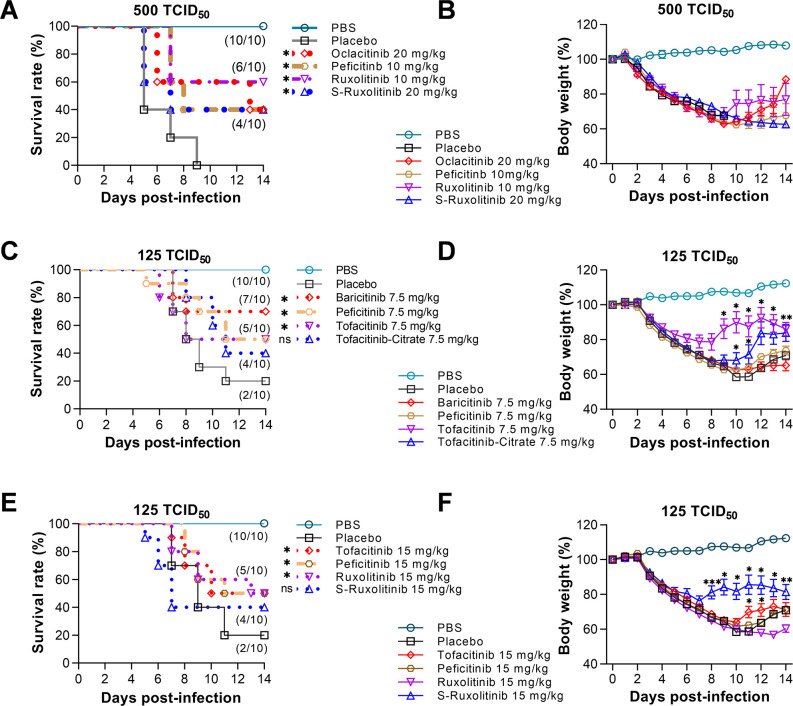
JAK inhibitors exhibit therapeutic effects on mice infected with the lethal influenza virus. (**A and B**) The percentage of survival and body weight of mice treated with four inhibitors (ruxolitinib, peficitinib, oclacitinib, and s-ruxolitinib) at 10 mg/kg and 20 mg/kg were measured in mice infected with 500 TCID_50_/50 µL PR8 virus. (**C–F**) Measurement of the percentage of survival and body weight of mice treated with six inhibitors (baricitinib, peficitinib, tofacitinib, tofacitinib-citrate, ruxolitinib, and s-ruxolitinib) at two different doses (7.5 mg/kg and 15 mg/kg) in mice infected with 125 TCID_50_ PR8 virus. Different groups of mice (10 mice per group) were treated with different inhibitors twice daily for 5 days starting at day 3 p.i., respectively. The survival rate figures did not display the efficacy of inhibitors with a survival rate of less than 40%. Significance in (A, C, and E) was determined using the log-rank test. Significance in (B, D, and F) was calculated with two-way ANOVA (analysis of variances) with Sidak’s post-test. *P < 0.05, **P < 0.01, and ***P < 0.001; ns, not significant.

To evaluate the therapeutic efficacy of these hit JAK inhibitors *in vivo*, we tested 10 inhibitors, which cover JAK1, 2, and 3 and have better anti-inflammatory properties (bold in Table 1, and the structures shown in Fig. 1), in a mouse influenza model. We inoculated the mice with 500 TCID50 of the PR8 virus, which caused 100% mortality in the infected mice. At day 3 post-infection (p.i.), the mice (10 in each group) were orally administered 10 or 20 mg/kg of each JAK inhibitor. All the mice were treated twice daily for 5 days. In addition, all mice were monitored daily for 14 days for weight loss and survival. During the experiment, the body weight of the mice treated with the placebo gradually decreased, and all mice died within 9 days p.i (dpi). However, mice treated with ruxolitinib at 10 mg/kg or peficitinib at 10 mg/kg survived at a rate of 60% and 40%, respectively. The ruxolitinib- and peficitinib-treated mice gradually regained body weight at day 10 p.i. When the drug dose was increased to 20 mg/kg, another two inhibitors, oclacitinib and s-ruxolitinib, were demonstrated to protect 40% of the infected mice (Fig. 2A and B). Notably, oclacitinib protected 60% of the infected mice until day 13, suggesting it is slightly better than s-ruxolitinib in the therapeutic effect.

Considering that a viral dose of 500 TCID50 of PR8 is too high for most JAK inhibitors, we reduced the viral amount to 125 TCID50, which resulted in an 80% mortality rate in infected mice. Our results showed that 7.5 mg/kg of baricitinib, peficitinib/tofacitinib, and tofacitinib-citrate were able to protect 70%, 50%, and 40% of the infected mice, respectively (Fig. 2C). The recovery of body weight in tofacitinib- and tofacitinib-citrate-treated mice started earlier than in baricitinib- or peficitinib-treated mice (Fig. 2D). We increased the dose of the drug to 15 mg/kg. We found that peficitinib and two additional inhibitors, ruxolitinib and s-ruxolitinib, protected 50%, 50%, and 40% of the infected mice, respectively (Fig. 2E). The body weights of s-ruxolitinib-treated mice recovered earlier and more efficiently than ruxolitinib-treated mice (Fig. 2F).

Therefore, in the primary *in vivo* efficacy study, 7 out of 10 JAK inhibitors showed protection against lethal influenza virus infection in mice, suggesting the potential of JAK inhibitors as anti-inflammatory agents.

### The dosage and efficacy of oclacitinib in the treatment of lethal influenza virus infection

**Fig 3:**
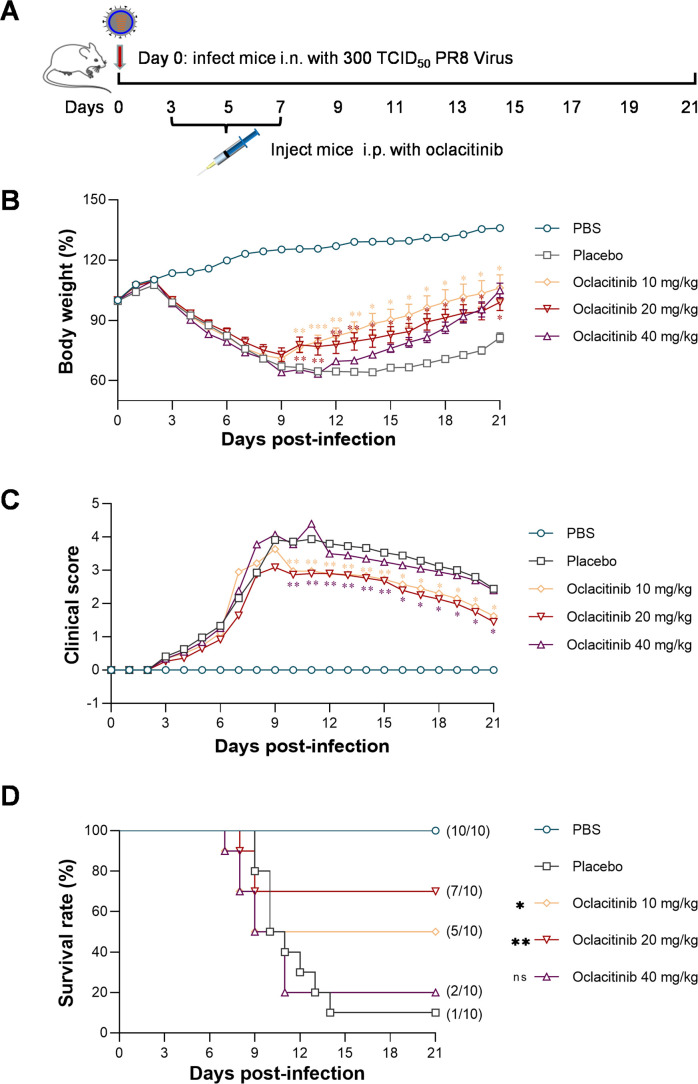
Oclacitinib’s dose and efficacy in treating mice infected with a lethal influenza virus. (**A**) Schematic diagram of the oclacitinib treatment of mice infected with influenza A virus. The experiments involved infecting different groups of mice (with 10 mice in each group) with 300 TCID_50_/50 µL PR8 virus and treating them with oclacitinib (at dosages of 10, 20, and 40 mg/kg) starting at day 3 p.i. for 5 days. The body weight loss percentage (**B**), clinical score (**C**), and survival rate (**D**) were monitored daily for 21 days. The results’ significance in weight loss and clinical scores was calculated using a two-way ANOVA with Sidak’s post hoc test. In contrast, the significance of survival was determined using a log-rank test. *P < 0.05, **P < 0.01, and ***P < 0.001 indicate a significant result, while ns indicates not significant.

To further investigate the *in vivo* therapeutic potential of JAK inhibitors for severe influenza-associated inflammation, we selected oclacitinib as a representative due to its high water solubility and ease of formulation. To determine the optimal dosage of oclacitinib, we conducted an *in vivo* efficacy study on BALB/c mice. Our previous study has shown that administering JAK inhibitors after the onset of influenza virus infection improves protection in BALB/c mice (22). We treated intraperitoneally five groups of BALB/c mice (10 in each group) infected with 300 TCID50 influenza viruses with varying doses of oclacitinib 3 dpi. One group was given a placebo, while the other three groups were intraperitoneally administered 40 mg/kg/d, 20 mg/kg/d, and 10 mg/kg/d doses of oclacitinib, respectively. A final group received an equal volume of phosphate-buffered saline (PBS) as the mock-infected control. Over 21 days, all mice were monitored for survival, weight loss, and clinical scores (Fig. 3A).

All infected mice experienced a decrease in body weight, which was at its lowest on day 9 p.i. However, mice treated with oclacitinib at dosages of 40 mg/kg/d, 20 mg/kg/d, and 10 mg/kg/d started to gain body weight. In contrast, as shown in Fig. 3B, the body weight of mice treated with placebo remained decreasing. Although the mice treated with 20 mg/kg/d oclacitinib showed mild clinical symptoms as indicated by clinical scores, it was the most effective treatment. On day 9 p.i., the clinical score of the mice treated with 20 mg/kg/d oclacitinib was the lowest among all the groups, as shown in Fig. 3C. The survival rate of mice treated with 20 mg/kg/d oclacitinib was significantly higher (*P* < 0.01) than that of placebo-treated mice. Seventy percent of the mice treated with 20 mg/kg/d oclacitinib survived, compared to only 10% of the placebo-treated mice. Even at a lower dosage of 10 mg/kg/d, oclacitinib still protected half of the infected mice population (50%), which was significantly higher (*P* < 0.05) than that of the placebo-treated mice (10%). However, there was no significant difference in the survival rates of mice treated with 40 mg/kg/d oclacitinib (20%) and placebo (10%), as shown in Fig. 3D. These findings suggest that oclacitinib is highly effective in protecting mice from fatal influenza virus infections. The optimal dosage in the experimental setting is 20 mg/kg/d.

### Oclacitinib suppresses neutrophils and macrophage infiltration and ameliorates lung injury in mice infected with lethal influenza virus

**Fig 4:**
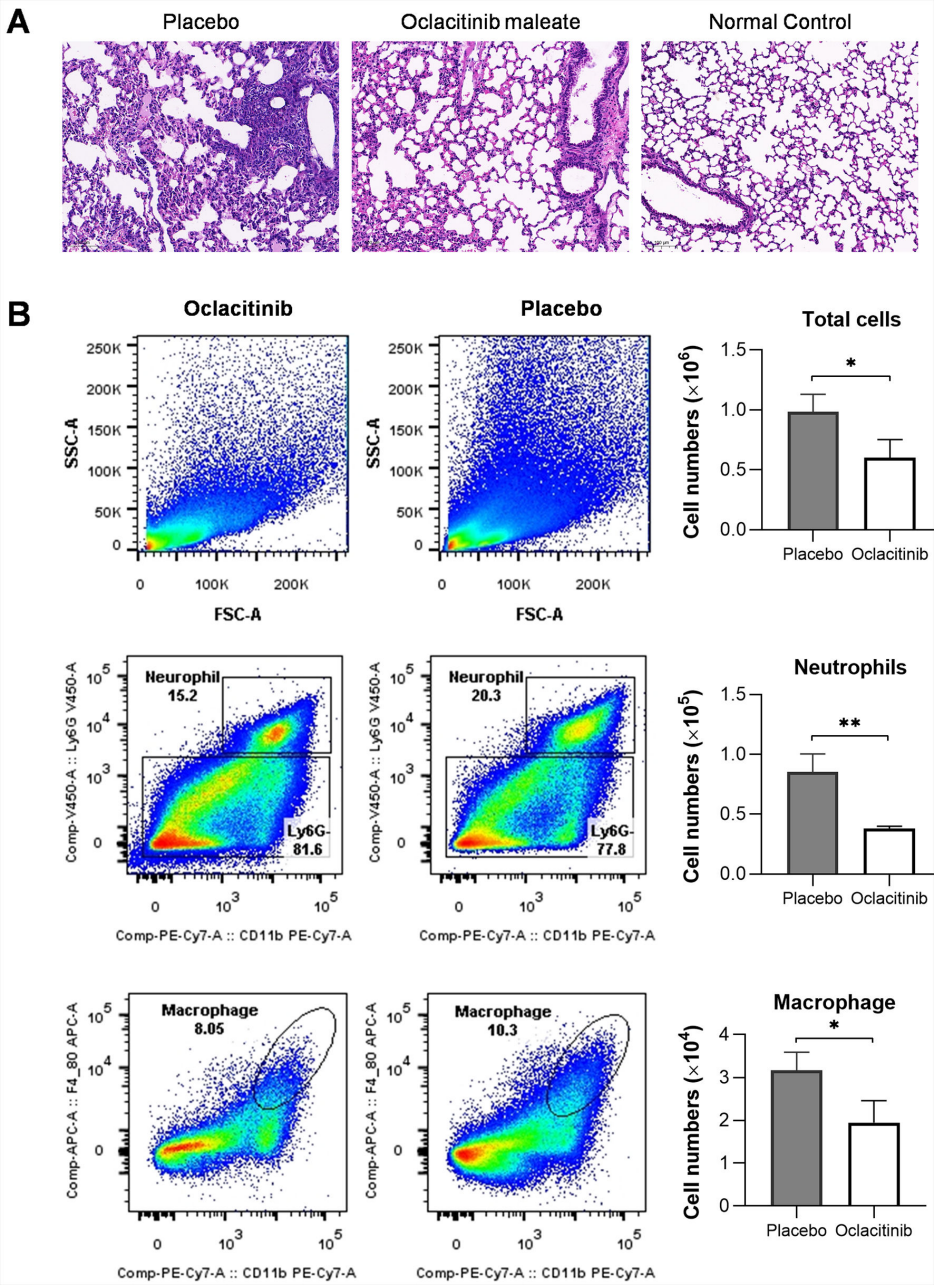
Oclacitinib suppresses neutrophil and macrophage infiltration and alleviates lung histopathology in mice infected with influenza viruses. (**A**) Two groups of mice, each consisting of three mice, were infected with 300 TCID_50_/50 µL PR8 viruses. One group was treated with oclacitinib (20 mg/kg/d) twice daily for 5 days starting at day 3 p.i. The other group was given a placebo. The third group was mock-infected and untreated as normal control. All the mice were euthanized on day 7 p.i. Their lung tissues were isolated and examined using histopathology. The lung sections of the mice treated with oclacitinib, placebo, and normal control mice were stained with hematoxylin and eosin (scale bar 100 µm). (**B**) Three groups of mice were treated as in (**A**). All the mice were euthanized on day 7 p.i., and their tracheas were isolated and injected with 1 mL PBS. Massage the thorax of the mouse and gently aspirate the solution. After centrifugation at 1,000 × g for 10 minutes at 4°C, the cell pellet was resuspended with 100 µL PBS containing 1% BSA (bovine serum albumin) and treated with Red Cell Lysis Buffer to lyse the red blood cells. After centrifugation of the solution at 1,000 × g for 10 minutes at 4°C, the cell pellet was resuspended and stained for further analysis in flow cytometry. The total cells, neutrophils, and macrophages in the BALF were counted and compared between the oclacitinib- and placebo-treated groups. The mean values ± SD are presented, and the statistical significance is indicated as *P < 0.05 and **P < 0.01.

To determine the impact of oclacitinib on the infiltration of inflammatory cells, we treated the mice with either a placebo or oclacitinib starting on day 3 p.i. The mice were euthanized on day 7 p.i. The lung tissues and bronchoalveolar lavage fluid (BALF) were analyzed using histopathology and flow cytometry. Based on HE (hematoxylin and eosin) staining results in Fig. 4A, the lung tissues of mice who received a placebo were severely damaged. There was extensive cell death, and large numbers of inflammatory cells were present in multiple areas. The accumulation of these cells caused the walls of the air sacs to thicken, and fluid was in the space between the sacs.

On the other hand, the lung tissues of mice treated with oclacitinib remained primarily intact, with fewer inflammatory cells present. The cells lining the airways were slightly damaged and shedding. The lungs of mock-infected mice showed no observable damage. Additionally, oclacitinib was found to reduce the number of neutrophils and macrophages in the lungs, determined by flow cytometry of the mouse BALFs, which are known to contribute to acute lung injury in influenza pneumonia (Fig. 4B) (23). Overall, oclacitinib effectively reduced inflammation by reducing neutrophils and macrophage infiltration in the lungs of infected mice.

### Oclacitinib reduces the production of pro-inflammatory cytokines/chemokines in mice with severe influenza but does not affect virus reproduction

**Fig 5:**
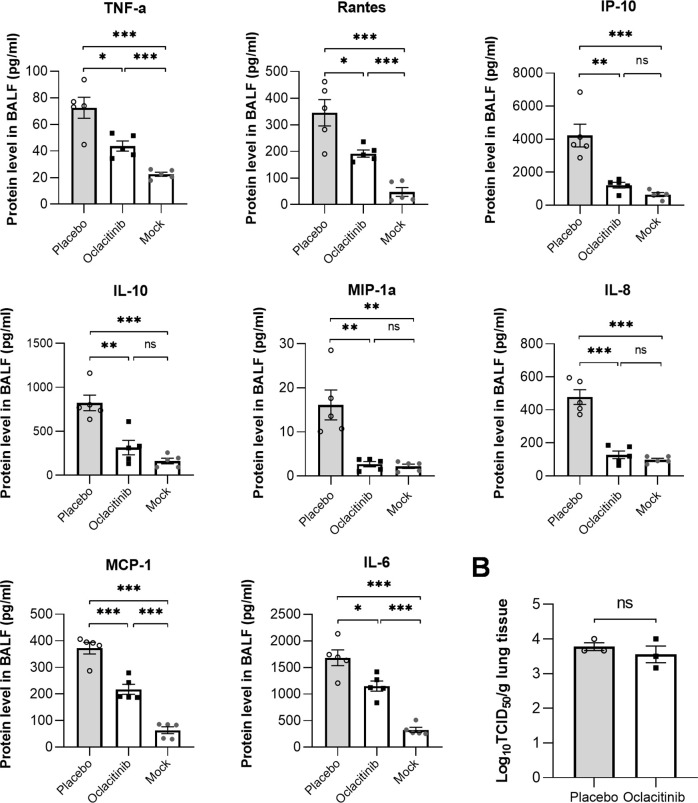
Oclacitinib inhibits the production of pro-inflammatory cytokines and chemokines in the lungs of mice infected with the lethal influenza virus but does not affect the viral titers. (**A**) Two groups of mice, each consisting of five mice, were infected with 300 TCID_50_/50 µL PR8 viruses. One group was treated with oclacitinib (20 mg/kg/d) twice daily for 5 days starting at day 3 p.i. The other group was treated with a placebo as virus control. The third group was mock-infected as normal control. All the mice were euthanized on day 7 p.i., and their BALF was prepared and used to measure cytokine production. Protein levels of pro-inflammatory cytokines and chemokines in BALFs of oclacitinib-, placebo-treated, or normal control mice (5 mice per group) kits were measured using enzyme-linked immunosorbent assay (ELISA) kits at day 7 p.i. (**B**) Viral titers in BALFs taken on day 7 p.i. were measured by TCID_50_ assay using Madin-Darby canine kidney (MDCK) cells (three mice per group). The data are representative of two independent experiments. *P < 0.05, **P < 0.01, and ***P < 0.001; ns, not significant.

Infection and rapid replication of the influenza virus in mice activate multiple signaling pathways, including JAK-STAT, to produce a large amount of pro-inflammatory cytokines and chemokines known as “cytokine storm,” which may cause severe pneumonia or ARDS. To further investigate the anti-inflammatory effects of oclacitinib, we measured the protein levels of eight cytokines and chemokines, which are essential in the immune response of influenza-infected mice, in BALFs prepared from oclacitinib- or placebo-treated mice at day 7 p.i. As shown in Fig. 5A, the production of all eight cytokines and chemokines, including TNF-α, RANTES, IP-10, IL-10, MIP-1α, IL-8, MCP-1, and IL-6, was significantly reduced in BALFs of oclacitinib-treated mice. Despite this reduction, there were no significant differences in viral titers between the treated and untreated mice in the BALF (Fig. 5B). These findings indicate that the effectiveness of oclacitinib treatment for influenza pneumonia in mice is solely due to its anti-inflammatory properties.

Thus, oclacitinib can reduce inflammation and protect mice from lethal influenza virus infection when used in the mouse influenza model. This effect may be due to its ability to lower the expression of pro-inflammatory cytokines and chemokines, reducing the infiltration of immune cells and lung damage.

### Treatment with oclacitinib suppresses the antibody response in mice to influenza virus infection

JAK inhibitors have been demonstrated to mitigate the inflammatory response to influenza infection in mice; however, they may also impact the adaptive immune response in these animals. Studies have shown that treatment with tofacitinib, a JAK inhibitor, or genetic knockout of JAK3 can effectively suppress antibody responses against protein antigens, leading to significant reductions in titers of IgG and IgM antibodies. Additionally, this intervention results in decreased numbers of pro-B cells and germinal center B cells, ultimately impairing the formation of germinal centers in mice (24). Recent research has unveiled that patients diagnosed with rheumatoid arthritis and treated with JAK inhibitors exhibit a suppressed humoral response subsequent to BNT162b2 (mRNA-based COVID-19 vaccines) vaccination. This is substantiated by the quantity and quality of the anti-spike antibodies (25). Furthermore, individuals suffering from immune-mediated inflammatory diseases, whose immune systems are compromised due to treatment with immunosuppressive medications, demonstrate a diminished response compared to healthy controls when subjected to the standard vaccination regimen (26, 27).

**Fig 6:**
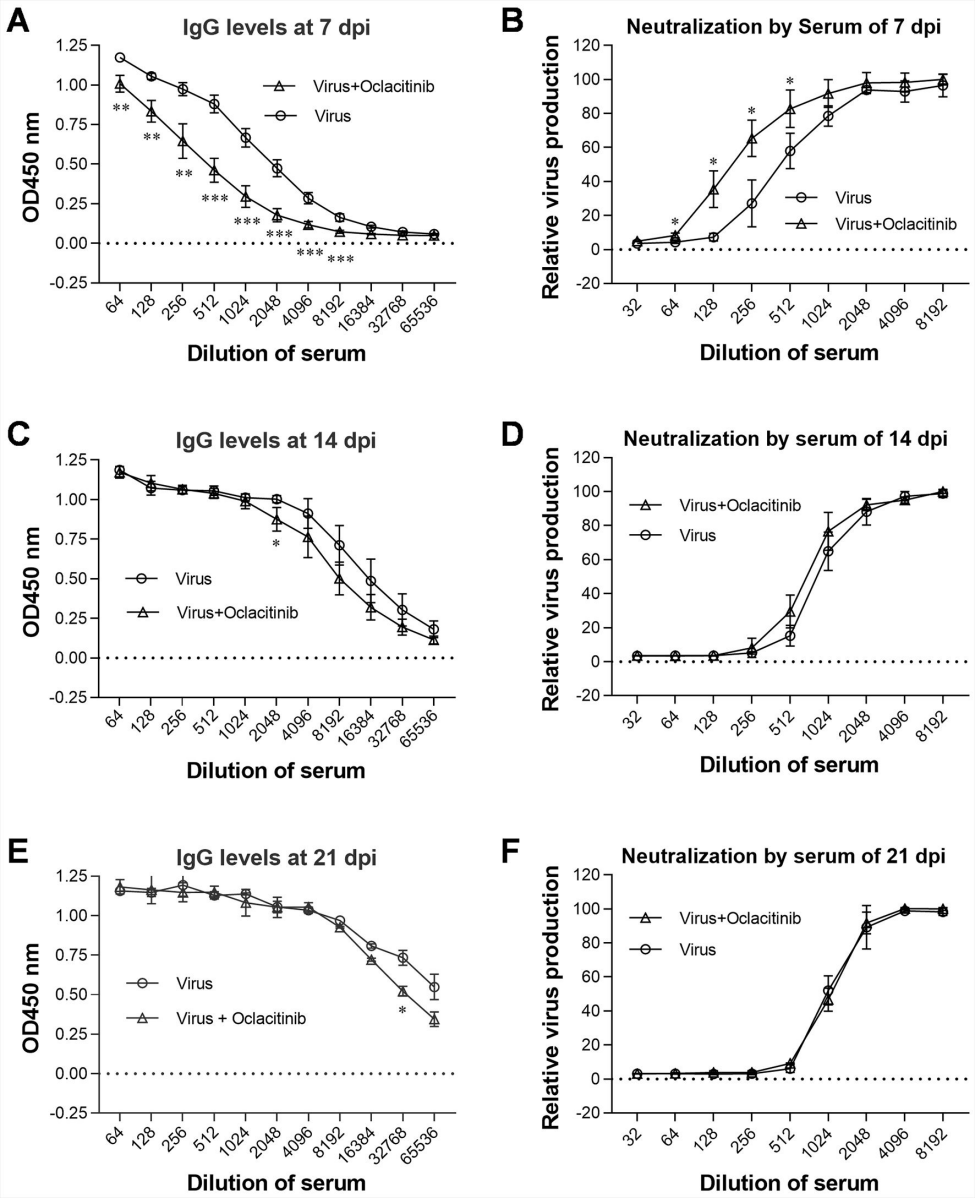
During the drug administration stage in PR8-infected mice, treatment with oclacitinib resulted in a reduction of both PR8-specific IgG and neutralizing antibody levels. Two groups of BALB/c mice (*n* = 15 per group) were inoculated with 50 TCID50 of the PR8 virus. One group received treatment with oclacitinib at a dosage of 20 mg/kg, administered twice daily for 5 consecutive days starting from day 3 p.i. The other group remained untreated. Sera were collected from three mice in each group at 7, 14, and 21 dpi, respectively. Influenza virus-specific IgG levels in the sera collected at these time points were determined using ELISA by analyzing serial dilutions of each serum sample (Fig. 6A, C and E). Neutralizing antibody titers in the sera collected at 7, 14, and 21 dpi from both the untreated and oclacitinib-treated mice were determined by neutralization assay through analysis of serial dilutions of each serum sample (Fig. B, D, and F). The mean values ± SD presented here represent two independent experiments. Statistical significance was assessed using two-way ANOVA (*P < 0.05; **P < 0.01; ***P < 0.001).

To investigate the impact of JAK inhibitor Oclacitinib on the antibody response to PR8 infection in mice, we inoculated two groups of BALB/c mice (15 per group) with 50 TCID50 of PR8 virus (which causes about 30%–40% of mouse death). One group received treatment with 20 mg/kg oclacitinib starting from 3 dpi, administered twice daily for 5 consecutive days. The other group remained untreated. Sera were collected from three mice in each group at 7, 14, and 21 dpi. Influenza virus-specific IgG levels were determined using enzyme-linked immunosorbent assay (ELISA) by analyzing serial dilutions of each serum sample (Fig. 6A, C and E). All sera exhibited influenza virus-specific IgG antibodies which increased over time following viral infection. Compared to the untreated mice, oclacitinib-treated mice showed significantly suppressed serum IgG production at 7 dpi and slight suppression at both 14 and 21 dpi.

Additionally, neutralizing antibody titers were measured in the sera; titers for the virus group were found to be 431, 863, and 1,015 at 7, 14, and 21 dpi, respectively, while those for the oclacitinib-treated group were observed as 177, 690, and 1,087 at 7, 14, and 21 dpi, respectively (Fig. 6B, D and F). These results demonstrate that neutralizing antibody titers increased over time from days 7 to 21 in both groups of mice; however, the oclacitinib-treated mice exhibited a threefold reduction in neutralization antibody titers compared to untreated mice at 7 dpi, demonstrating a slight decrease at 14 dpi but no difference by day 21 dpi.

Overall, oclacitinib treatment significantly impaired the antibody response to influenza virus infection during administration; however, recovery was observed after cessation of treatment.

## DISCUSSION

Severe influenza is caused by excessive inflammatory response to highly replicated influenza viruses (28). Traditional anti-inflammatory drugs, such as steroids, are ineffective or have severe side effects (18, 19, 29). Meanwhile, non-steroidal anti-inflammatory drugs are inadequate for treating severe pneumonia caused by highly pathogenic influenza viruses (30). However, newer immunomodulators, such as monoclonal antibodies and small molecular inhibitors with new host targets, have recently been developed. For example, tocilizumab, a humanized anti-IL6 receptor antibody, has successfully treated rheumatoid arthritis, juvenile idiopathic arthritis, and Castleman disease (31) and has also shown promise in treating a COVID-19 patient with respiratory failure (32). JAK inhibitors like baricitinib and ruxolitinib have also been shown to reduce recovery time and improve clinical symptoms in COVID-19 patients (33, 34). These studies suggest that new classes of anti-inflammatory agents with minimal side effects and new host targets, like JAKs, are promising for developing anti-inflammatory drugs for severe influenza virus infections.

We have identified 15 JAK inhibitors that effectively inhibit the production of three pro-inflammatory cytokines induced by influenza virus infection following two rounds of screening. Among all the anti-inflammatory drugs we examined, JAK inhibitors exhibited a higher hit rate compared to compounds targeting alternative targets. Our analysis revealed that the selected JAK inhibitors demonstrated significant suppression of each cytokine IP-10, IL-8, and MCP-1 with high SIs, most exceeding 1,000, as presented in Table 1. This is not surprising since the influenza virus infection triggers the expression of a large quantity of interferon α/β, which activates JAK-STAT signaling pathways and induces the expression of many pro-inflammatory cytokines/chemokines. Our findings suggest that JAK-STAT signaling pathways play a crucial role in excessive inflammation caused by influenza virus infection. Therefore, JAK inhibitors could be a promising therapy for treating influenza pneumonia.

Our study evaluated the efficacy of 10 JAK inhibitors in treating influenza pneumonia in mice infected with a lethal influenza virus. Out of the tested 10 JAK inhibitors, seven were found to protect a significant portion of the infected mice, ranging from 40% to 70%. However, the three JAK inhibitors with higher cytotoxicity—AZ960, AZD1480, and gandotinib (Table 1)—did not protect against the lethal infection. This inefficiency could be due to the toxicity of these drugs at the given dosages. It is important to note that the efficacy of a medicine can be affected by various factors such as dosage, formulation, and route of administration. Therefore, we anticipate better efficacy with most JAK inhibitors in further optimization and trials. Our primary *in vivo* experiments suggest that JAK inhibitors have great potential for treating influenza pneumonia with anti-inflammatory monotherapy.

Further *in vivo* experiments indicated that oclacitinib reduced pro-inflammatory cytokine production, suppressed inflammatory cell infiltration, and finally ameliorated the lung injury of the infected mice. Notably, the progeny viral titer was unaffected by oclacitinib treatment, suggesting that the therapeutic effect of oclacitinib is due to its anti-inflammatory activity, which makes it a promising candidate for clinical therapies targeting inflammation induced by influenza virus infection.

We studied the effect of JAK inhibitor treatment on the antibody response of mice to influenza virus infection. Our results indicate that while oclacitinib administration initially suppressed the antibody response, there was a subsequent recovery in the production of influenza virus-specific IgG and neutralization antibodies after cessation of treatment. Patients who do not take immunosuppressive medications, such as JAK inhibitors, are unlikely to have compromised adaptive immune responses since anti-inflammatory therapy is short-term. However, patients who take long-term immunosuppressive medications may experience impaired or weakened adaptive immune responses.

Among the JAK inhibitors that offer protection against lethal influenza virus infection, ruxolitinib and tofacitinib are JAK inhibitors that offer protection against autoimmune diseases (35–38). Baricitinib is an FDA-approved oral JAK inhibitor that improves clinical symptoms of rheumatoid arthritis and is also used in COVID-19 patients (39, 40). These inhibitors have been found to have minimal side effects even when used for an extended period of time (41–43). As the anti-inflammatory therapy for influenza pneumonia is short-term, it is considered safe to use JAK inhibitors, especially those that have passed safety tests in clinical trials. These drugs hold great promise for treating severe cases of influenza in humans.

Although the exact mechanisms of anti-inflammatory therapy for influenza pneumonia are not yet fully understood, our research suggests that JAK-STAT signaling pathways are potential anti-inflammatory drug targets, and JAK inhibitors have great potential to be a new and safe class of anti-inflammatory agents for treating influenza pneumonia.

## MATERIALS AND METHODS

### Cell lines, animals, and virus strains

Madin-Darby canine kidney (MDCK) cells (ATCC CCL-34^™^) were maintained in Dulbecco’s modified Eagle’s medium (DMEM, Gibco, Carlsbad, CA) supplemented with 10% fetal bovine serum (Gibco), 100 U/mL penicillin, and 100 µg/mL streptomycin. Human monocyte cell line U937 (CRL-1593.2) was cultured in RPMI 1640 medium supplemented with 10% fetal bovine serum (Gibco), 100 U/mL penicillin, and 100 µg/mL streptomycin. All these cells were maintained at 37℃ in a 5% CO_2_ incubator. Influenza virus strain A/Puerto Rico/8/1934 (H1N1) was provided by the Wuhan Institute of Virology, Chinese Academy of Sciences. The viral titers were measured using 50% tissue culture infective dose (TCID_50_) assay in MDCK cells. The 10-day-old embryonated chicken eggs were obtained from Guangdong Wens Dahuanong Biotechnology Co., Ltd (Guangdong, China). Six-week-old BALB/c mice were purchased from Guangdong Medical Laboratory Animal Center (GDMLAC, Guangdong, China) and housed under specific pathogen-free conditions.

### Chemicals

The 15 JAK inhibitors (Table 1) were purchased from Selleck Chemicals (Houston, TX, USA). Each inhibitor was dissolved in DMSO (dimethyl sulphoxide) and stored at −80°C for later use.

### U937 cell-based anti-inflammatory assay for influenza virus infection

U937 cells were seeded in a 96-well culture plate at a density of 1.5 × 10^5^ cells per well and incubated for 24 hours prior to virus infection. Subsequently, the cells in each well were infected with the PR8 virus at a multiplicity of infection of 0.05 and treated with serially diluted drugs or left untreated as a control. The drugs were serially diluted threefold, starting from an initial concentration of 200 µM down to 0.01 µM. All cell cultures were maintained in RPMI 1,640 medium supplemented with 1% FBS and incubated at 37 ℃ under a CO_2_ atmosphere (5%) for 72 hours. Following this incubation, the supernatants were collected and subjected to AlphaLISA analysis to determine IL-8, IP-10, and MCP1 protein levels.

### Enzyme-linked immunosorbent assay (ELISA) and AlphaLISAy

Mouse IL-10 (Cat. No. 431414), IL-6 (Cat. No. 431304), and TNF-α (Cat. No. 430901) ELISA kits were obtained from BioLegend (San Diego, USA). Mouse IL-8 (CME0008), MCP-1 (CME0046), and IP-10 (CME0051) ELISA kits were purchased from 4A Biotech (Beijing, China). Mouse CCL3/MIP-1α (EK261/2-96) ELISA kit was bought from Multi Sciences (Hangzhou, China). Mouse CCL5/RANTES (BSEM-048–96T) ELISA kit was obtained from Biosharp (Hefei, China). The ELISA was conducted by following the manufacturer’s instructions.

The AlphaLISA detection kits employed in this study were IL-8 (PerkinElmer AL224), IP-10 (PerkinElmer AL259), and MCP1 (PerkinElmer, AL244). U937 cell culture supernatants were measured for IL-8, IP-10, and MCP-1 protein levels using AlphaLISA assay as per the manufacturer’s recommendations. The ELISA and AlphaLISA plates were read using the Varioskan LUX multimode microplate reader (Thermo Scientiﬁc, Waltham, MA, USA).

### Cytotoxicity assay

The compound cytotoxicity was determined by CellTiter-Glo Assay (Promega, Madison, WI, USA). After adding 15 µL of CellTiter-Glo reagent to each well, plates were incubated at room temperature for 15 minutes with shaking. The luminescence intensity was determined by a multimode plate reader (Wallac Envision, PerkinElmer, MA, USA).

### Animal experiments

Female BALB/c mice (6-week-old) were anesthetized by isoflurane and then intranasally infected with the mouse-adapted influenza virus A/Puerto Rico/8/1934 (H1N1; 500 or 125 TCID_50_ diluted in PBS) in a volume of 50 µL. The mice in the mock group were inoculated with an equal volume of PBS. In the primary evaluation, all the 10 inhibitors, including oclacitinib maleate, peficitinib, tofacitinib citrate, s-ruxolitinib, baricitinib, tofacitinib, AZ960, gandotinib, AZD1480, and ruxolitinib were dissolved in DMSO and then resuspended in 0.9% sodium chloride containing 0.5% CMC-Na, 30% PEG300, and 5% tween 80 separately. Each inhibitor was administered orally twice daily for 5 consecutive days. Following infection, all the mice were monitored daily for their survival, weight loss, and clinical signs of illness for 14 days. Each mouse was assigned a daily clinical score, ranging from 0 to 5, using the standards modified from previously described (44). Briefly, the scoring criteria were as follows: 0 = no visible signs of illness; 1 = slight fur ruffling; 2 = fur ruffling, reduced mobility, and decreased body weight; 3 = fur ruffling, reduced mobility, decreased body weight, and rapid breathing; 4 = fur ruffling, minimal mobility, huddled appearance, rapid and labored breathing, and decreased body weight; 5 = death or moribund.

To further evaluate the *in vivo* efficacy, oclacitinib maleate was dissolved in 0.9% sodium chloride for the dosages of 10, 20, and 40 mg/kg/day and administered intraperitoneally twice daily for 5 consecutive days. Female BALB/c mice were infected with the influenza virus A/Puerto Rico/8/1934 (H1N1) (300 TCID50 diluted in PBS) in a volume of 50 µL. The mice were monitored daily for their survival, weight loss, and clinical signs of illness for 21 days. On day 7 p.i., the mice were sacrificed. Their tracheas and lungs were removed and washed twice by injecting 1 mL PBS containing 0.1% BSA. After centrifugation at 1,000 × g for 10 minutes at 4°C, the BALF was collected and used for further analysis.

### Preparation of BALF

The mouse was euthanized and affixed onto a surgical plate, followed by disinfection of the neck using 70% ethanol. An incision was made on the neck skin using scissors to expose the trachea. Subsequently, a 0.2 cm incision was created on the trachea with surgical scissors, and a gavage needle was carefully inserted into it. The trachea was tied around the needle using cotton thread to secure the needle in place. Then, sterile PBS solution loaded in a 1 mL syringe was gently injected into the lung. The thorax of the mouse was massaged to facilitate even distribution of the solution before careful aspiration. Following the removal of the syringe from the needle, lavage fluid obtained from this process was transferred to an ice-cooled 1.5 mL tube for further processing or stored at −80℃ for subsequent analysis. Finally, resuspension and staining of cell pellet obtained after centrifugation of lavage fluid for 10 minutes at 1,000 × g and 4℃ were performed for subsequent analysis using flow cytometry (45).

### Flow cytometry

BALF samples were washed with FACS (fluorescence-activated cell sorting) buffer (2% BSA in PBS), followed by red blood cell lysis using Red Cell Lysis Buffer (Tiangen, Beijing, China). For the surface staining, cells were stained with the following fluorochrome-labeled antibodies (Biolegend, San Diego, USA): PE-Cy7-conjugated Ly6C (clone HK1.4, dilution 1:200), PE-conjugated CD11b (clone M1/70, dilution 1: 200), APC-conjugated Ly6G (clone 1A8, dilution 1: 200), and FITC-conjugated CD11c (clone N418, dilution 1: 100). Cells were washed with PBS twice and counterstained with 7AAD (Biolegend, San Diego, USA) to differentiate apoptotic and dead cells, then analyzed using a BD FACSVerse Cell Analyzer (BD, New Jersey, USA).

### Lung tissues histopathology

The H&E staining experiments were performed according to the previous report (46). Briefly, all the mouse lung tissues were fixed in 4% paraformaldehyde overnight, embedded in paraffin, and cut into 4 µm sections. The sections were stained with hematoxylin solution, treated with hematoxylin differentiation solution and hematoxylin Scott’s tap water bluing, and rinsed with tap water. The sections were dehydrated in 85% ethanol for 5 minutes, followed by 5 minutes in 95% ethanol, and stained with eosin dye for 5 minutes. After dehydration, all the sections were sealed with neutral gum. Each section was examined under the microscope. The slides were randomly read and reviewed for tissue damage, necrosis, and inflammatory cell infiltration.

### Determination of influenza virus-specific IgG in mouse serum

The PR8 virus was purified and inactivated using β-Propiolactone (CAS: 57–57-8, MCE) at a 1:3,000 (vol/vol) ratio. The inactivated PR8 virus (2 µg in each well) was then coated onto a 96-well ELISA plate (Corning, USA) and left overnight at 4℃. Afterward, the plate was washed three times and blocked with 5% skimmed milk for 1 hour at 37℃. The plate was washed again three times and then incubated with 100 µL of serial diluted sera at 37 ℃ for 2 hours. Next, the plate was washed three times and incubated with 100 µL of 1,000-fold diluted (horseradish peroxidase) HRP-conjugated affinipure goat ant-mouse IgG (H + L; SA00001-1, Proteintech) at 37℃ for 1 hour. After washing the plate four times, 100 µL of TMB (3, 3', 5, 5'-tetramethylbenzidine) substrate was added to each well until the solutions turned blue, and then 50 µL of 2M H_2_SO_4_ was added to stop the reaction. Finally, the absorbance was read at OD450 nm using the Varioskan LUX multimode microplate reader (Thermo Scientific, Waltham, MA, USA) (47).

### Neutralization assay

The neutralization assay was performed to determine the neutralizing titers of sera obtained from mice infected with influenza virus. The procedure involved diluting the sera and subsequently heat-inactivating it at 56℃ for 30 minutes, followed by its combination with 100 TCID_50_ PR8 virus and incubation at 37℃ for 1 hour. The mixture was added to pre-seeded MDCK cells and incubated at 37℃ for 1 hour. Subsequently, the medium was replaced with fresh DMEM and further cultured at 37℃ for 24 hours. Virus production in the supernatant was quantified by assessing virus neuraminidase activity on a black opaque 96-well plate (PerkinElmer, 6005270) using a substrate of 20 µM 2'-(4-methylumbelliferyl)-α-D-N-acetylneuraminic acid (MUNANA, Sigma, M8639) dissolved in MES solution [33 mmol/L 2-(N-morpholino) ethanesulfonic acid and 4 mmol/L CaCl_2_, pH = 6.5]. The reaction mixture was then incubated at 37℃ for an additional period of 2 hours. The reaction was terminated by the addition of a stop solution (0.14 mol/L NaOH in 83% ethanol). Fluorescence intensity was measured using multimode plate readers (Varioskan Lux, Thermoscientific, USA) with an excitation wavelength of 355 nm and an emission wavelength of 485 nm. The relative virus production was determined based on the fluorescence intensity, which correlated to the cleavage of MUNANA by viral neuraminidase. The serum neutralization titer, representing the dilution fold of serum required to achieve 50% virus neutralization, was determined using Graphpad software (47).

### Statistical analyses

The concentrations required to inhibit cytokine production by 50% (IC_50_), reduce cell viability by 50% (CC_50_), and SIs (which is equal to CC_50_/IC_50_) of compounds were calculated using Prism v8.0 software (GraphPad Software, San Diego, CA). Data were presented as mean ± SEM for each point. Differences between averages between control samples and tests were statistically analyzed using Student’s *t* test. *P*-values < 0.05 were considered statistically significant. Two-way repeated measures ANOVA with post-hoc Bonferroni *t* test was performed for body weight studies. Survival of mice was compared using the Log-Rank (Mantel-Cox) test.
